# The GSTome Reflects the Chemical Environment of White-Rot Fungi

**DOI:** 10.1371/journal.pone.0137083

**Published:** 2015-10-01

**Authors:** Aurélie Deroy, Fanny Saiag, Zineb Kebbi-Benkeder, Nassim Touahri, Arnaud Hecker, Mélanie Morel-Rouhier, Francis Colin, Stephane Dumarcay, Philippe Gérardin, Eric Gelhaye

**Affiliations:** 1 Université de Lorraine, Interactions Arbres—Microorganismes, UMR1136, F-54500, Vandoeuvre-lès-Nancy, France; 2 INRA, Interactions Arbres—Microorganismes, UMR1136, F-54280, Champenoux, France; 3 Laboratoire d’Etudes et de Recherches sur le Matériau Bois, EA4370 Université de Lorraine USC INRA, Faculté des Sciences et Technologies, 54506, Vandoeuvre-les-Nancy, France; 4 AgroParisTech, UMR 1092 LERFOB, F-54000, Nancy, France; 5 INRA, UMR 1092 LERFOB, F-54280, Champenoux, France; University Of Helsinki, FINLAND

## Abstract

White-rot fungi possess the unique ability to degrade and mineralize all the different components of wood. In other respects, wood durability, among other factors, is due to the presence of extractives that are potential antimicrobial molecules. To cope with these molecules, wood decay fungi have developed a complex detoxification network including glutathione transferases (GST). The interactions between GSTs from two white-rot fungi, *Trametes versicolor* and *Phanerochaete chrysosporium*, and an environmental library of wood extracts have been studied. The results demonstrate that the specificity of these interactions is closely related to the chemical composition of the extracts in accordance with the tree species and their localization inside the wood (sapwood vs heartwood vs knotwood). These data suggest that the fungal GSTome could reflect the chemical environment encountered by these fungi during wood degradation and could be a way to study their adaptation to their way of life.

## Introduction

Specialized fungi are the major actors of wood microbial degradation in forest ecosystems. Among them, the white-rot fungi are the only known organisms able to mineralize all the different components of this recalcitrant substrate. This unique ability is due to the development of complex enzymatic and non-enzymatic extracellular systems allowing oxidative and hydrolytic degradation of the different wood components [1, 2, 3]. This way of life requires also large intracellular networks involved in detoxification/catabolism processes. The extracellular oxidative degradation generates indeed a myriad of potentially toxic compounds that the fungus has to deal with. Furthermore, these fungi have also to cope with specific components of wood, called wood extractives, which are part of the tree defense system [4].

The wood decaying fungi possess also extended intracellular detoxification systems [5]. This intracellular system is composed in particular of a large number of glutathione transferases (GSTs), enzymes involved in the second phase of the detoxification processes. These enzymes allow both an increase of the solubility and a decrease of the chemical reactivity of potential toxic molecules. The number of genes encoding GSTs, is usually largely superior in wood-decayer genomes in comparison with other fungi [6]. This high number of GSTs is mainly due to the extension of specific phylogenetically based classes named Ure2p, GST Omega (GSTO) and GSTFuA [7, 8].

These classes have been differentiated from a phylogenetic point of view but their specific function remain to be elucidate. Furthermore, the classes responsible for the observed extension of GST encoding genes differ from one fungus to another. For instance, in *Trametes versicolor*, nearly half of the total GSTs belong to the GSTO class (16/38), whereas in *Phanerochaete chrysosporium*, one third belongs to the Ure2p class (9/27) [6].

To date, each characterized fungal phylogenetic class exhibits particular structural features [9–11]. Phylogenetically, the Ure2p class could be split into two subclasses, named Ure2pA and Ure2pB. The Ure2pA subclass is fungal specific and is found extended in various wood decaying fungi. Members of the Ure2p class characterized so far possess the ability to bind oxidized glutathione [8]. Few studies have been devoted to fungal GST related to the omega class. Among this class, members possess either a cysteinyl or a serinyl residue in their catalytic site. The proteins harboring a cysteinyl residue exhibit a deglutathionylation activity whereas the presence of a serinyl residue seems to be associated with a glutathione transferase activity of the corresponding proteins [11, 12].

From these studies devoted to GSTs from wood-decaying fungi, it appears that the different GSTs characterized so far share usually a low specificity whatever their class and organism origin. They display indeed usually the same kind of catalytic (glutathione transferase, thiol transferase, peroxidase, esterase…) and ligandin (*i*.*e*. ability to bind ligands) activities against more or less artificial substrates/ligands [7, 12].

From our previous data on fungal GSTs, we hypothesize that the study of the GST network found in white-rot fungi could give insights on their adaptation to different wood species, and could reflect at least partly the chemical environment encountered by these fungi during wood degradation [6, 7]. To test this hypothesis, we investigated the biochemical interactions of 6 GSTOs from *Trametes versicolor* and 5 Ure2ps from *Phanerochaete chrysosporium* with an environmental collection of wood extracts [13]. These both GST classes (i.e. GSTO and Ure2p) are the most extended in the studied fungi [6]. The obtained data demonstrate a close relationship between these interactions and the chemical composition of the tested extractives.

## Materials and Methods

### Expression and purification of proteins

Among the 16 GSTO genes found in the genome of *Trametes versicolor*, 8 have been selected in the most extended subclass III [12], all of them exhibiting a serinyl residue in their putative catalytic site (accession number in the JGI database: TvGSTO-1S: Tv75639; TvGSTO-2S: Tv56280; TvGSTO-3S: Tv48691; TvGSTO-4S: Tv65402; TvGSTO-5S: Tv54358 and TvGSTO-6S: Tv23671). Recombinant plasmids were obtained by synthetic gene synthesis, cloning into the NdeI and NotI restriction site of pet 26b plasmid. The sequences have been designed to add a C-term His-tag for purification (S1 Table). Expression was performed at 37°C using the transformed *Escherichia coli* Rosetta2 (DE3) pLysS strain (Novagen) in LB medium containing kanamycin (50 μg·ml−1) and chloramphenicol (34 μg·ml−1). When the cell culture reached an optical density at 600 nm of 0.7, protein expression was induced with 0.1 mM IPTG and cells were grown for a further 4 h. Cells were harvested by centrifugation, resuspended in a 30 mM Tris/HCl (pH 8.0) and 200 mM NaCl lysis buffer and stored at −20°C. Cell lysis was completed by sonication (three times for 1 min with intervals of 1 min). The cell extract was then centrifuged at 35000 ***g*** for 25 min at 4°C to remove cellular debris and aggregated proteins. 10 mM imidazole was added in the soluble fraction before loading on to an Ni-NTA (Ni^2+^-nitrilotriacetate)–agarose resin connected to an ÄKTA purifier FPLC system (GE Healthcare). After a washing step with lysis buffer supplemented with 20 mM imidazole, the proteins were eluted using an imidazole gradient from 20 to 500 mM. The fractions of interest were pooled, dialysed, concentrated by ultrafiltration under nitrogen pressure (YM10 membrane; Amicon), and stored in 30 mM Tris/HCl (pH 8.0) and 200 mM NaCl buffer at −20°C.

The homogeneity of the proteins was checked by SDS/PAGE. Protein concentration was determined by measuring the absorbance at 280 nm and using theoretical molar absorption coefficients of 38390 M^−1^·cm^−1^, 29972 M^−1^·cm^−1^, 35410 M^−1^·cm^−1^, 46410 M^−1^·cm^−1^, 42462,5 M^−1^·cm^−1^, 26930 M^−1^·cm^−1^ deduced from the primary sequences of TvGSTO-1S, TvGSTO-2S, TvGSTO-3S, TvGSTO-4S, TvGSTO-5S, TvGSTO-6S respectively.

Concerning Ure2p from *Phanerochaete chrysosporium*, five proteins, one belonging to the subclass B named PcUre2pB1 and four to the subclass A named PcUre2pA4, PcUre2pA5, PcUre2pA6 and PcUre2pA8 have been studied. The production and the partial characterization of these proteins have been previously described [8].

### Wood extracts

For competition assays 60 extractives were obtained from knotwood and stemwood of different tree species. Norway spruce [*Picea abies* (L.)], European larch [*Larix decidua Mill*], cherry [*Prunus avium L*.] and beech [*Fagus sylvatica L*.] were sampled in the Amance forest near Nancy (northeast France) in winter 2012–2013. Walnut [*Juglans regia L*.], cedar [*Cedrus atlantica Manetti*], metasequoia [*Metasequoïa glyptostroboides*] were harvested in a private nursery in Laneuvelotte and chestnut [*Castanea sativa Miller*] was felled in Saverne forest (both in northeast France). Evergreen oak [*Quercus ilex L*.] was harvested near Aix-en-Provence (southeast France) and maritime pine [*Pinus pinaster Aiton*] was sampled in INRA domain near Orléans (northwest France). These trees were felled during winter 2013–2014. Trees come from the National Forest Service ONF and AgroParisTech, private nursery Adam et Fils (48°43'30.15"N 6°17'20.12"E), M. Lopez from IRSTEA Aix-en-Provence and INRA Orléans. We confirm that the field studies did not involve endangered or protected species.

On every trunk, a disc was sawn at a height of 1.3 m; inner and outer parts were sampled to isolate heartwood and sapwood, respectively. Several discs were sawn above the previous one containing either dead or living knots, which were recovered using a wood chisel. All samples were dried at 50°C until mass stabilization. For some species, when knots amounts were too small, dead and living knots were mixed all together while for the other cases, they remained separated. Heartwood, sapwood and knots samples were then ground to fine sawdust with particle size between 0.2 to 0.4mm before extraction. Each powder was Soxhlet-extracted using acetone. A defatting step with methylene chloride was performed before the acetone extraction [14], except for the extracts named global in the Table 1. After each extraction organic solvents were evaporated under vacuum using a rotary evaporator. Dried extractives were stored in a freezer before GC-MS analyses.

**Table 1:** Main compounds and relative composition of stemwood and knotwood acetone extracts.

	Fatty Acids	Gallic Acids	Resin Acids	Sugars	Others	Unidentifie Flavonoids	Catechin	Taxifolin	Sakuranin	Naringenin	Dihydrochrysin	Others Lignans	Secoisolariciresinol	Matairesinol	Unidentified
**Beech heartwood**	6.3														93.7
**Beech sapwood**	29.9				16.9										53.2
**Beech mixed knots** ^**b**^				73.4	0.7		2.0								23.9
**Beech mixed knots** ^**b**^ **global** ^**a**^	11.2			63.3	5.2										20.3
**Cherry heartwood**					4.9	18.8			24.3		29				23.0
**Cherry heartwood global** ^**a**^			2.7	1.8	4.0	22.7	2.9		12.5	40.7					12.7
**Cherry sapwood**	5.7		8.5			12.1	51.8								21.9
**Cherry mixed knots** ^**b**^				8.3		26.6	47.5								17.6
**Cherry mixed knots** ^**b**^ **global** ^**a**^				6.6	0.2	30.7	33.6								28.9
**Chestnut heartwood**		60.5		24.7	6.4	1.6									6.8
**Chestnut sapwood**	0.8			94.3	4.3										0.6
**Chestnut dead knots**		20.8		79.2											
**Chestnut living knots**		56.3		43.7											
**E.oak heartwood**				83.9			13.2								2.9
**E.oak sapwood**				97.1			2.9								
**E.oak dead knots**	6.9	10.5		16.1	5.6		52								8.9
**E.oak living knots**				74.4			24.2								1.4
**Larch heartwood**					4.5	37.6									57.9
**Larch heartwood global** ^**a**^	1.3		15.5	5.3	1.2	60.9									15.8
**Larch sapwood**	14.4		59.3		6.6										19.7
**Larch mixed knots** ^**b**^				4.0	1.2	4.2		45.3				14	22.5		8.8
**Larch mixed knots** ^**b**^ **global** ^**a**^			7.7					38				5.4	30.3	11	7.6
**Walnut heartwood**		2.9		55.2	22.5		19.4								
**Walnut sapwood**				90.1	9.9										
**Walnut living knots**				96.4	2.3										1.3

^a^ Global means.

^b^ Mixed knots means that living and dead knots were mixed when ground and extracted

### Chemical characterization of the wood extracts

Gas chromatography coupled to mass spectrometer (GC–MS) allowed the identification and the relative quantification of the different substances present in the wood extracts (Table 1). Samples were analyzed as trimethylsilyl derivatives using the following procedure. In a screw-capped vial, a sample of about 1mg of dry extract was dissolved in 100μL of BSTFA/TMCS 1%. The solution was vortex-stirred for about 1 min and heated at 70°C for 20hrs. After evaporation of the silylating reagent, the residue was diluted in 1 mL of ethyl acetate. The GC–MS analysis was performed on a Clarus 600 GC gas chromatograph coupled to a SQ8 mass spectrometer (Perkin-Elmer). Separation was carried out on a 5% diphenyl/95% dimethyl polysiloxane fused-silica capillary column (J&W Scientific DB-5MS, 30m×0.25mm×0.25μm). The injection was performed at 250°C in the splitless mode with Helium as carrier gas, at a constant flow of 1mL.min^-1^. Chromatographic conditions were as follows: initial temperature 80°C, 2min isothermal, 10°C.min^-^1 to 190°C, 15°C.min^-^1 to 280°C, 10min isothermal, 10°C.min^-^1 to 300°C, 14min isothermal. The components ionization was performed by electron impact (70eV ionization energy) to achieve their identification by mass spectra comparison with the NIST Library. Samples relative composition in extractives was obtained by the area of each peak determined on the TIC (Total Ion Current) chromatogram divided by the sum of all the detected peaks areas.

### Fluorescence-based thermal stability assay

This assay was adapted from Boivin et al (2013), allowing following protein denaturation [15]. Thermal denaturation exposes hydrophobic region of proteins, which could interfere with the SYPRO orange dye, this latter being fluorescent in hydrophobic environment. The experimental procedure was performed in 96 well microplate (Harshell, Biorad) and the measurements were carried out with real time PCR detection system (CFX 96 touch, Biorad). The assays were achieved as follow: 5μL of Tris-HCl (150mM) pH8 buffer, 2μL of substrate (wood extract at an initial concentration of 10 mg/ml in DMSO), 2μL of proteins for a final concentration of 40μM, 2μL of SYPRO orange (Sigma) previously diluted 62 fold and 14μL of ultra pure water for a total volume of 25μL per well. The plate was centrifuged 30 seconds at 4000g. The fluorescence was measured (at excitation at 485nm and emission at 530nm) each minute starting with 3 minutes at 5°C and increasing temperature from 5° to 95°C with a step of 1°C per minute. The melting temperature (Tm), which corresponds to the temperature where the protein is 50% unfolded, was determined using the first derivative of the obtained data in presence/absence of potential ligands [15]. Wood extracts (stock solutions at 10 mg/ml in DMSO) have been used at a final concentration of 1mg/ml. Differences in melting temperatures (∆Tm) could also be calculated and normalized (see below).

### Competition experiments (CE)

The competition experiments were based on esterase activity displayed by the studied fungal GSTs. 5 chloromethylfluorescein diacetate (CMFDA) was used as fluorogenic substrate. These assays were performed on 96 wells microplates and measurements were carried out with Victor3 microplate reader (Wallac Parkin Elmer Life Sciences) at 535nm for emission and 485nm for excitation. A solution was prepared with 21mL of TE 30mM (30mM TrisHCl pH8, 0.1mM EDTA), 10.5mL of CMFDA at 100μM, 210μL of GSH 100mM and 210μL of protein. The protein concentration varied from one protein to another in order to obtain a constant rate during duration of the test. The assays were performed as follow: 199μL of the prepared solution was distributed in each well and a first measurement was carried out during 10 minutes which 1 measure per minute. Immediately after, 1μL of wood extract (stock solutions at 10 mg/ml in DMSO) was added in each well and the measurements were taken again during 60 minutes which 1 measure per minute. The linear increases of fluorescence were used for further analysis (see below).

### Glutathionylation assays

The glutathione transferase acitivity was measured following the glutathionylation of CDNB (1-chloro-2,4-dinitrobenzene) as described by Lallement et al (2014) [16].

#### Principal component analysis

For each protein, thermal shift experiments allowed the determination of melting temperatures (Tm) in presence of the different wood extracts. As reference, similar experiments were conducted adding only DMSO allowing the determination of Tm_ref_. The principal component analysis was performed on the normalized values T calculated as follow: T = (Tm- Tm_ref_)/ Tm_ref_.

Concerning the competition experiments performed using the CMFDA test; data used in the principal component analysis were calculated as follow. The slope (S) (linear increase of fluorescence determined during the first 10 min after addition of the product) was calculated, the value obtained only in presence of DMSO being the reference (Sr). The principal component analysis was performed using the normalized value I with I = (S/Sr).

Principal component analysis was implemented using the xlstat software (Pearson correlation). A matrix of the T and I values determined as described above for the different studied proteins were used as input (S2 Table)

## Results and Discussion

### Interactions between GSTs and wood extracts

To test our hypothesis that fungal GST network could give insights on the adaptation of white rot fungi to different wood species, we used GSTs belonging to expanded classes (omega and ure2p) in two different white-rot fungi, *Trametes versicolor* and *Phanerochaete chrysosporium*.

Genome mining of the sequenced *Trametes versicolor* has revealed the presence of 16 genes encoding putative GST Omega (GSTOs) [6]. Six TvGSTOs exhibiting a serinyl residue in their catalytic site were selected from the most extended subclass III [12] for this study. The glutathione transferase activity of the purified proteins (S1 Fig) has been tested using CDNB as substrate. All proteins exhibit an activity against CDNB, even if this activity remains low for different isoforms (kcat of 6 and 51 min^-1^ for TvGSTO-2 and TvGSTO-5S respectively). The kinetic parameters are reported in the S3 Table. These data are in agreement with previous results obtained on *Phanerochaete chrysosporium* isoforms showing that GSTO harboring a serinyl residue in their catalytic site exhibit a classical glutathione transferase activity [11, 12].

Concerning PcUre2ps, we used previously characterized Ure2ps from *Phanerochaete chrysosporium*, four isoforms belonging to the Ure2pA subclass (PcUre2pA4, 5, 6 and 8), and the unique member of the Ure2pB subclass in this fungus [8, 9]. In both cases, GSTO and Ure2p, these proteins belong to the most extended GST class in *Trametes versicolor* and *Phanerochaete chrysosporium* respectively.

Fungal GSTs as GSTs from other organisms are known to display both catalytic activities and ligandin activities (ability to bind/interact with ligands) [17]. Depending of the considered fungal isoforms, the site involved in the ligandin activity (site L) could be related to the glutathione binding site (site G), to the hydrophobic site involved in the substrate binding (site H) or to be completely independent of the G and H sites [10, 18, 19]. In this context, the investigation of the interactions between GST and chemical libraries is required to study both catalytic and ligandin activities.

To investigate the potential ligandin activities of the tested GSTs, we used a fluorescence-based thermal stability (FTS) approach with SYPRO Orange as fluorophore [20, 15]. This method is widely used to identify ligands of proteins [21, 22]. The influence on the catalytic activities could not be measured using the classical CDNB test, since the resulting glutathionylated product of the reaction is followed at 280 nm, limiting the screening of aromatic molecules. An alternative fluorogenic substrate (CMFDA) was then tested. This substrate is currently used to test the esterase activity of glutathione transferases [12]. The different studied enzymes (the 6 TvGSTO and the 5 PcUre2p) exhibit all an activity in presence of glutathione against this substrate allowing its use for further screening experiments.

**Fig 1:**
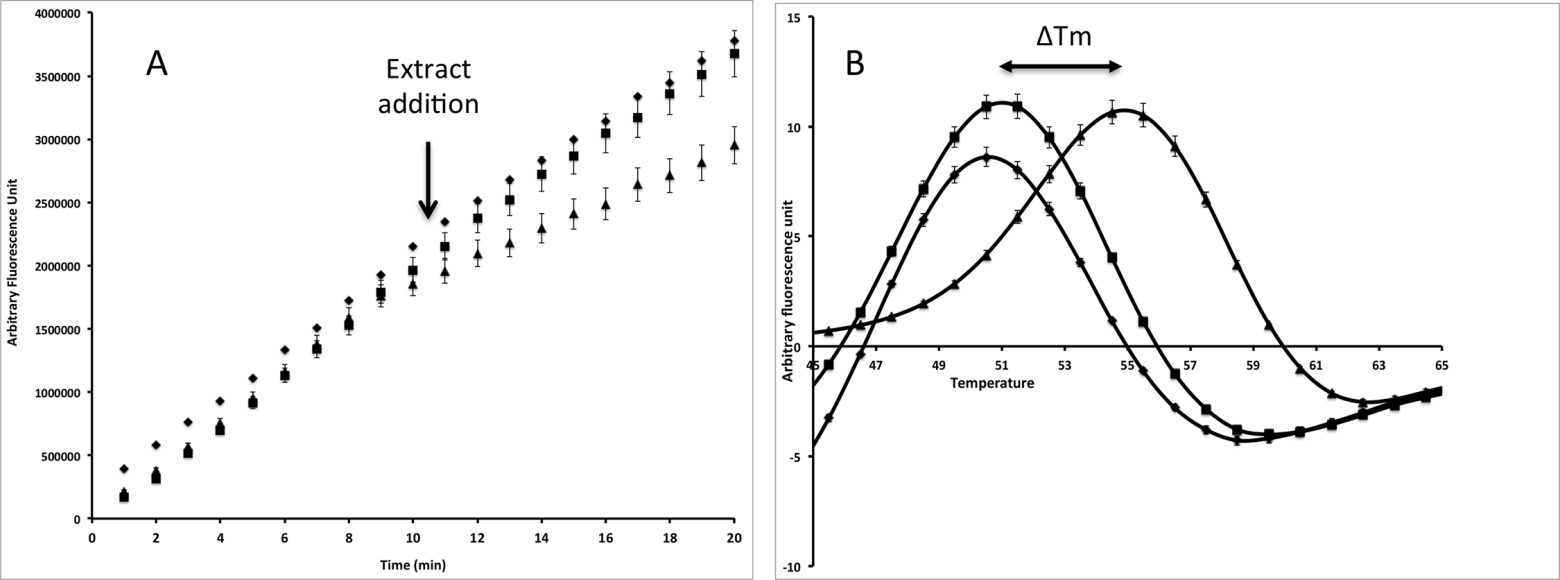
Effects of larch acetonic extracts on the catalytic activity (A) and thermal stability (B) of TvGSTO-1S. The catalytic activity was monitored following the cleavage of CMFDA by fluorescence as described in materials and methods section. DMSO or the tested extracts have been added after 10 min. For the thermal stability, the first derivatives of the raw data are shown allowing to determinate the Tm. diamond: DMSO (control); square: larch sapwood; triangle: larch heartwood

FTS and competition experiments using CMFDA were then combined to study the interactions between the studied GSTs and an environmental library composed of 60 different wood extracts. This library contained acetone and dichloromethane extracts of sapwood, heartwood and knotwood from different hardwood and softwood species (S4 Table). A shift on the protein melting temperature (Tm) revealed an interaction the tested product and the protein, whereas an inhibition of the CMFDA transformation revealed an interaction between the tested product and either the G site, or the H site or both. As an example, the effects of acetonic extracts from larch heartwood and sapwood on TvGSTO1-S catalytic and ligandin activities are shown in Fig 1. The heartwood extract induced both an inhibition of the catalytic activity (Fig 1A) and a shift of the measured Tm (Fig 1B), whereas no effects were observed in presence of sapwood extracts in comparison with the control (only DMSO).

**Fig 2:**
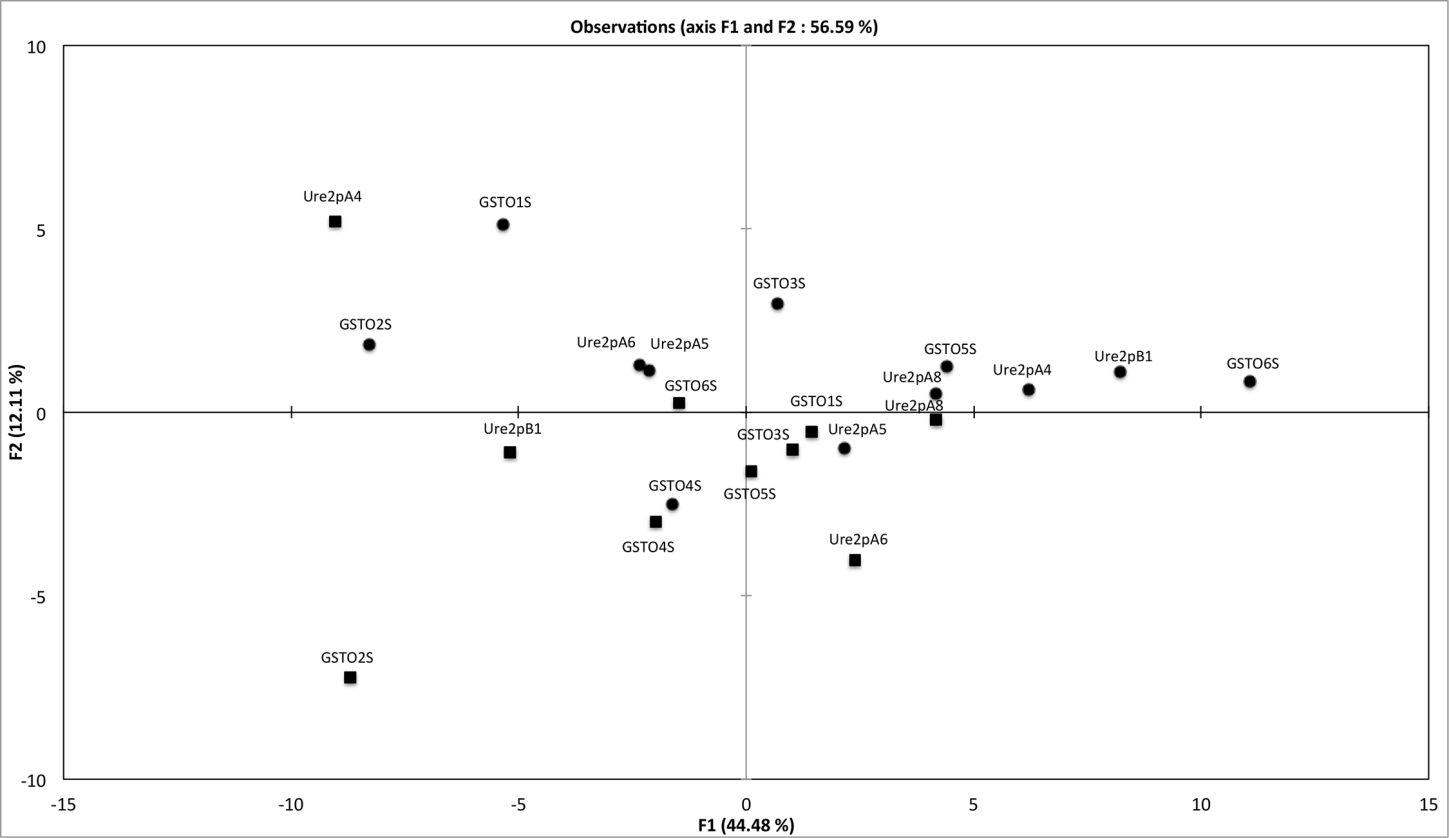
Principal component analysis plot showing the distribution of six GST Omega (GSTO) from *Trametes versicolor* and five Ure2p from *Phanerochaete chrysosporium*. A matrix based on the interactions, measured using the fluorescence-based thermal stability assay (squares) and the competition experiments (circle), between 60 wood extracts and these tested GSTs was used as input.

A principal components analysis (PCA) was implemented using the normalized obtained data from FTS and CE experiments as input (The used matrix is shown in S2 Table). The tested proteins were found widespread in the obtained biplot (PC1 and PC2 accounted of 56.59% of the total variance) without a clear clustering (Fig 2). Both approaches (FTS and CE) group some proteins as TvGSTO2S, TvGSTO4S or PcUre2pA8. In contrast, both methods gave different results for others such as PcUre2pB1 and TvGSTO6S for instance, suggesting that in this case ligandin (L site) and catalytic domains (G and/or H sites) are clearly different. These data confirm that FTS and CE approaches are complementary and their combination could be a way to investigate the relationship between the diversity of fungal GSTs and the variability of wood extracts.

### TvGSTOs and hardwood acetone extracts

**Fig 3:**
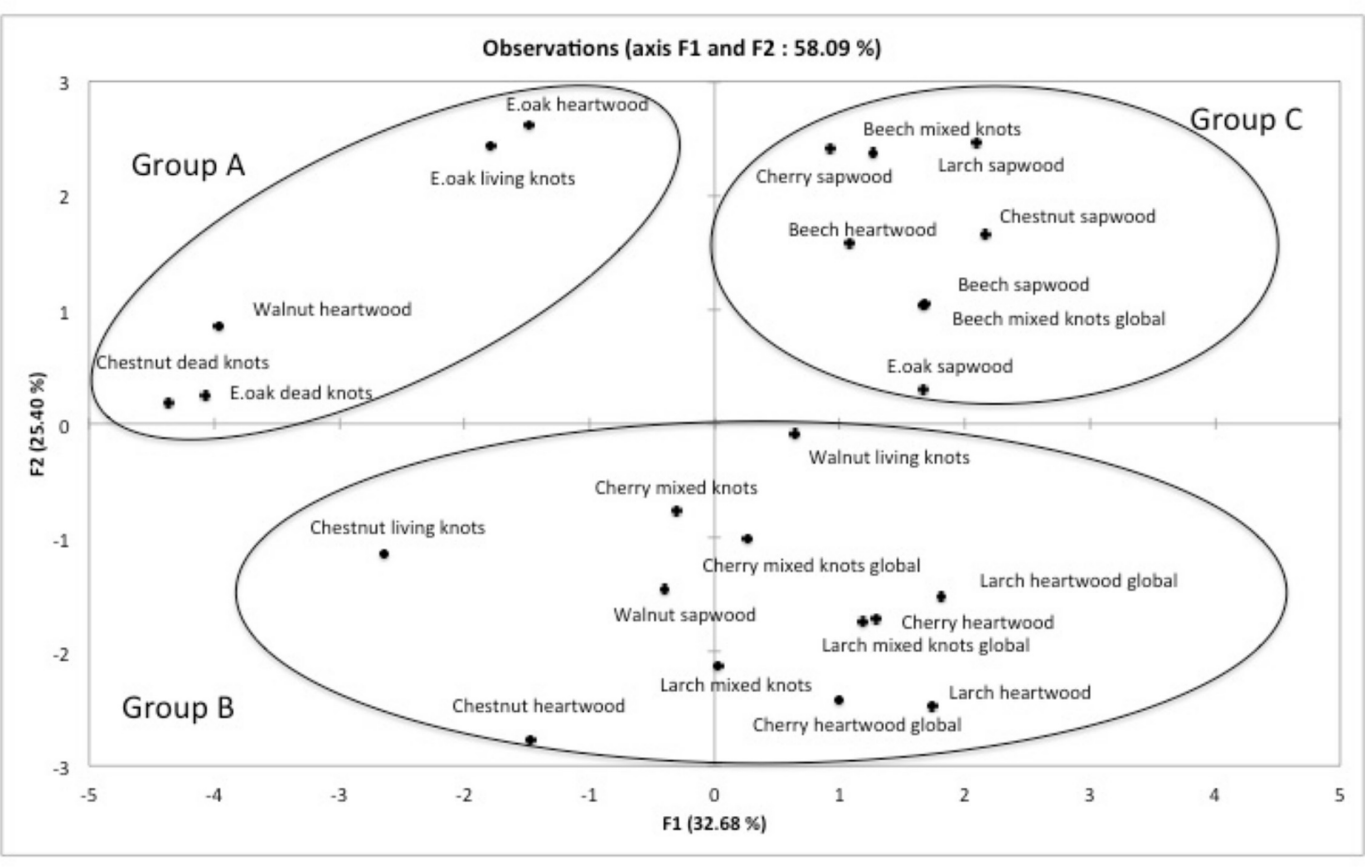
Principal component analysis plot showing the distribution of acetonic extractives from tested hardwood. A matrix based on the interactions between the six studied TvGSTO and these extractives determined using the fluorescence-based thermal stability assay and the competition experiments was used as input

Whereas the ecology of *P*. *chrysosporium* remains unclear, *T*. *versicolor* is a widespread fungus known as a prime wood decomposer of hardwood in temperate forests. The interactions between the six studied TvGSTOs and acetone extracts from several hardwoods and larch have also been specifically analyzed. Larch extracts have been integrated in this study since this softwood exhibits an extract composition similar to that one found in hardwoods [13]. The relative chemical composition of these extracts was determined using GC-MS analysis (Table 1). They contain different main classes of molecules as sugars, terpenes, lignans or flavonoids and also a more or less important uncharacterized fraction. PCA has been implemented on the data obtained after FTS and CE performed on TvGSTOs and chosen extracts (S2 and S4 Tables). PC1 and PC2 accounted for 58.09% of the total variance (Fig 3). The observed distribution into three groups is statistically significant as shown by the ANOVA performed on the obtained biplot coordinates (p<0.05). The first group (A) contains (i) chestnut and oak dead-knotwood, (ii) oak living-knotwood and also (iii) walnut and oak heartwood extracts. These extracts are mainly characterized by the exclusive presence of catechin among the flavonoids family and gallic acid. The second group (B) is characterized by the presence of mixture of flavonoids, gallic acid and lignans in the wood extracts. Extracts from larch heartwood and knotwood contain indeed a large proportion of uncharacterized flavonoids. This chemical composition is similar to that one observed in extracts of cherry heartwood and knotwood and could explain their close distribution. Nevertheless, it is important to mention that walnut sapwood, contains no flavonoid clusters in this group. A possible explanation could be the presence of chemicals also detected in heartwood but not fully identified. Finally the last group (C) contains sapwood extracts from most of the tested tree species (the only exception being walnut sapwood) and all beech extracts. These extracts are characterized by the absence (or a very low relative concentration) of flavonoids, gallic acid and lignans with the exception of cherry sapwood extract. This latter contains a high relative yield of uncharacterized flavonoids and belongs however to this last group. Analysis of the chemical composition highlights significant differences between the extracts belonging to the different groups. Presence/absence of gallic acid (Fisher analysis, p<0.0001) and presence/absence of flavonoids (Fisher analysis, p<0.005) could indeed explain at least partially the observed PCA distribution. From our results, it appears that the study of the biochemical interactions between GSTs and wood extracts could be a way to discriminate between these latter. This discrimination is largely correlated with their chemical composition. In particular, the obtained data suggest that the studied TvGSTOs could be used as enzymatic tools to discriminate wood extractives containing potential chemical active molecules such as gallic acid or flavonoids.

**Fig 4:**
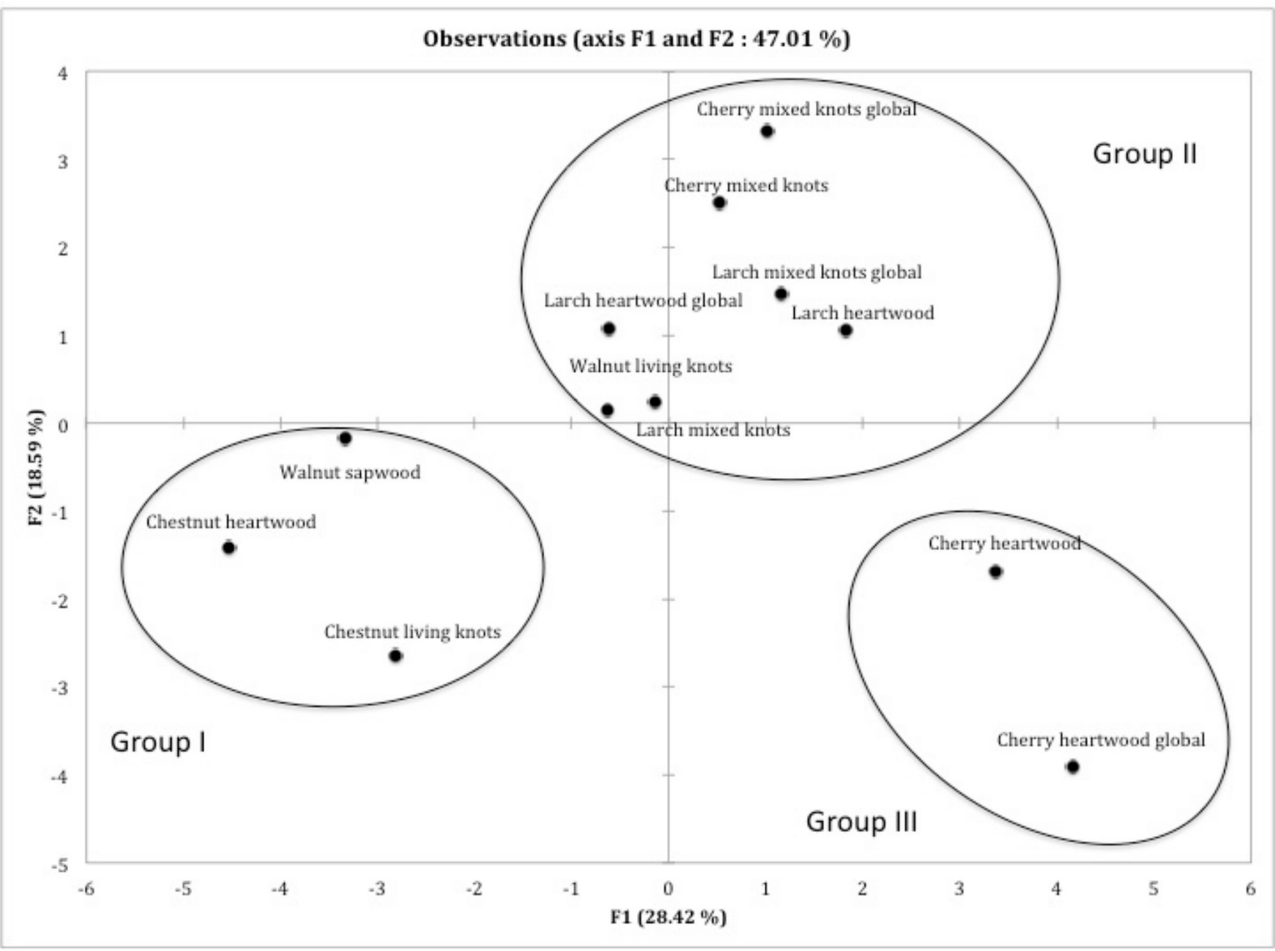
Principal component analysis plot showing the distribution of acetonic extractives of the group B defined in Fig 3. This group B is a chemically similar group of extractives based on major functional categorizations of chemicals found in the extracts. A matrix based on the interactions between the six studied TvGSTO and the five studied PcUre2p and these extractives determined using the fluorescence-based thermal stability assay and the competition experiments was used as input

In order to get a more accurate discrimination of the extracts belonging to the previously described group B, another PCA was implemented using FTS and CE data with both TvGSTO and PcUre2p proteins and the group B wood extracts as input. This PCA led to three sub-groups statistically distinct (Fisher analysis on the coordinates, p<0.0001), separating in particular clearly cherry knotwood from cherry heartwood extracts (Fig 4). The presence of sakuranin in cherry heartwood extracts (ANOVA performed on the chemical composition, p<0.0001), and more globally the difference in flavonoids composition could explain this repartition. In accordance the previous analysis (Fig 2), the presence of gallic acid could be related to the observed distribution of the chesnut extracts (p<0.01). To confirm that TvGSTOs and PcUre2ps could indeed interact with gallic acid and flavonoids, we tested the effects of pure compounds, (gallic acid and two flavonoids, epicatechin and quercetin) on the thermostability and the esterase activity of the different tested proteins. The obtained results demonstrate that globally TvGSTOs and PcUre2ps interact with these compounds (modification of the thermostability and inhibition of the esterase activity) (data not shown).

Wood decaying fungi have developed a complex network of GSTs, enzymes involved in detoxification processes. The low specificity of these enzymes suggests that they are able to interact with a large numbers of substrates/ligands [6, 7, 8]. In another hand, one major factor involved in natural durability of wood is the presence of “wood extractives”, which are potentially toxic for microorganisms [4]. We report here that the study of the biochemical interactions between GSTs and wood extracts could be a way to access to the diversity of these latter. These interactions are indeed closely related to the chemical composition of the wood extracts and could reflect in some cases the origin (tree species and location in wood) of these extracts. From these data, it appears in agreement with our working hypothesis, that the GST network found in white-rot fungi could indeed give insights on their adaptation to different wood species and also to their chemical environment,

## Supporting Information

S1 Fig SDS-PAGE showing the different steps of TvGSTO-2S protein purification. S = standard (250 kDa, 150 kDa, 100 kDa, 75 kDa, 50 kDa, 37 kDa, 25 kDa, 20 kDa, 15 kDa, 10 kDa)— = fraction without protein induction, TF = total fraction, IF = insoluble fraction, SF = soluble fraction, FT = flow through, W1 and W2 = wash, E = elution (protein).
(TIFF)

S1 Table Nucleotide sequences of TvGSTO. Sequences designed to add a C-term His-tag for purification. NdeI and NotI restriction site of pet 26b are underlined.
(TIFF)

S2 Table Normalized Data from fluorescence thermal shift assay and competition experiment used as input for principal component analysis. Assays have been performed as described in Materials and methods TvGTO-1S; TvGTO-2S; TvGTO-3S; TvGTO-4S; TvGTO-5S; TvGTO-6S and PcUre2pB1; PcUre2pA4; PcUre2pA5; PcUre2pA6 and PcUre2pA8.
(TIFF)

S3 Table Activity of the six GSTO from *Trametes versicolor* against CDNB. Catalytic parameters were determined using varying substrate concentrations at saturating GSH concentration by fitting the results to the Michaelis-Menten equation using GraphPad Prism 5 software. Data are represented as mean ± S.D (n = 3).
(TIFF)

S4 Table Library of different wood extracts. The Library contained acetone and dichloromethane extracts of sapwood, heartwood and knotwood from different hardwood and softwood species used for CE and FTS assays.
(TIFF)
