# Supplementary figures and images for: The GSTome Reflects the Chemical Environment of White-Rot Fungi
Source: PLoS One. 2015 Oct 1;10(10):e0137083. doi: 10.1371/journal.pone.0137083 (PMC4591263; doi:10.1371/journal.pone.0137083)

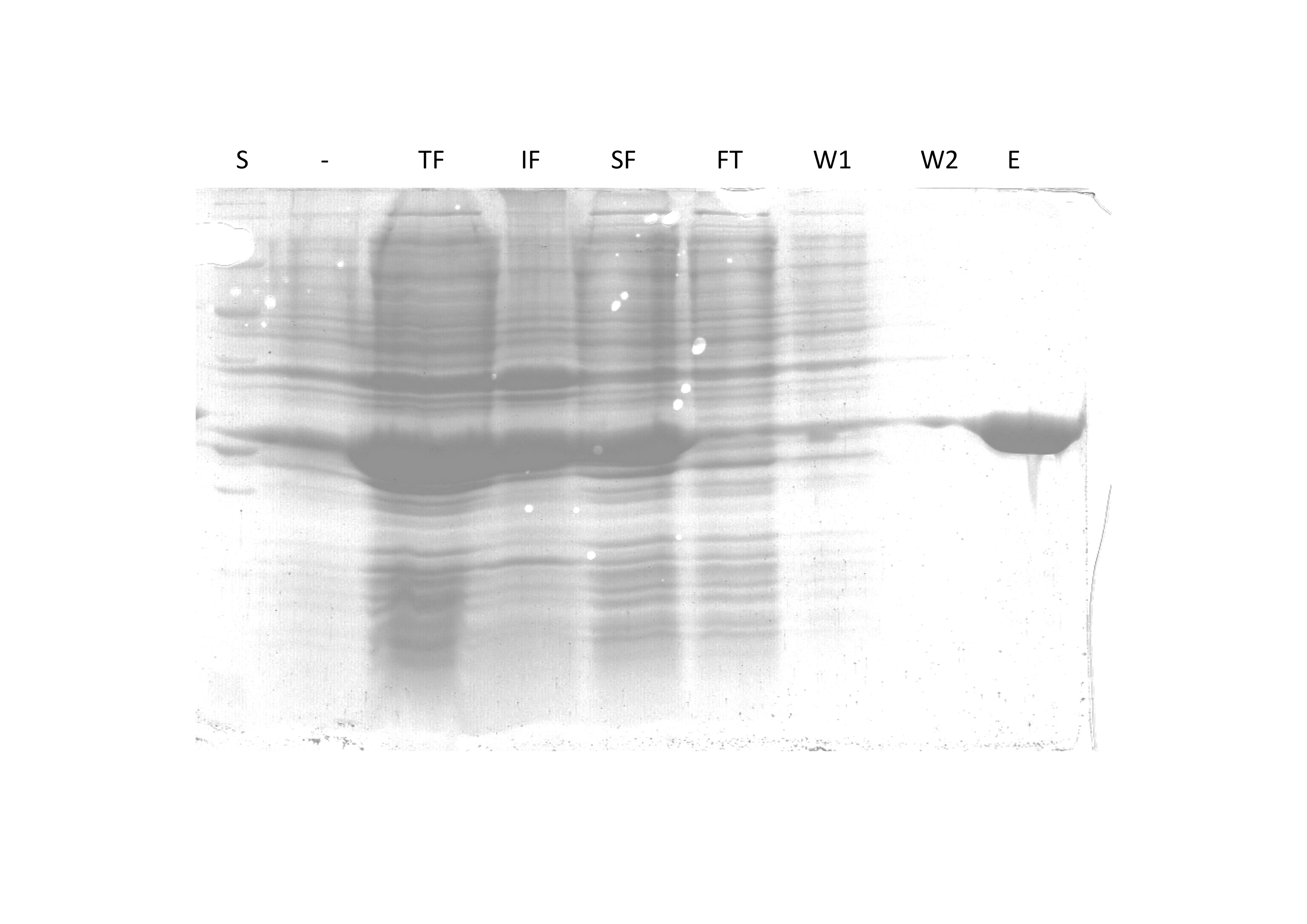

Supplement: S1 Fig — S = standard (250 kDa, 150 kDa, 100 kDa, 75 kDa, 50 kDa, 37 kDa, 25 kDa, 20 kDa, 15 kDa, 10 kDa)— = fraction without protein induction, TF = total fraction, IF = insoluble fraction, SF = soluble fraction, FT = flow through, W1 and W2 = wash, E = elution (protein). (TIFF) [file pone.0137083.s001.tiff]

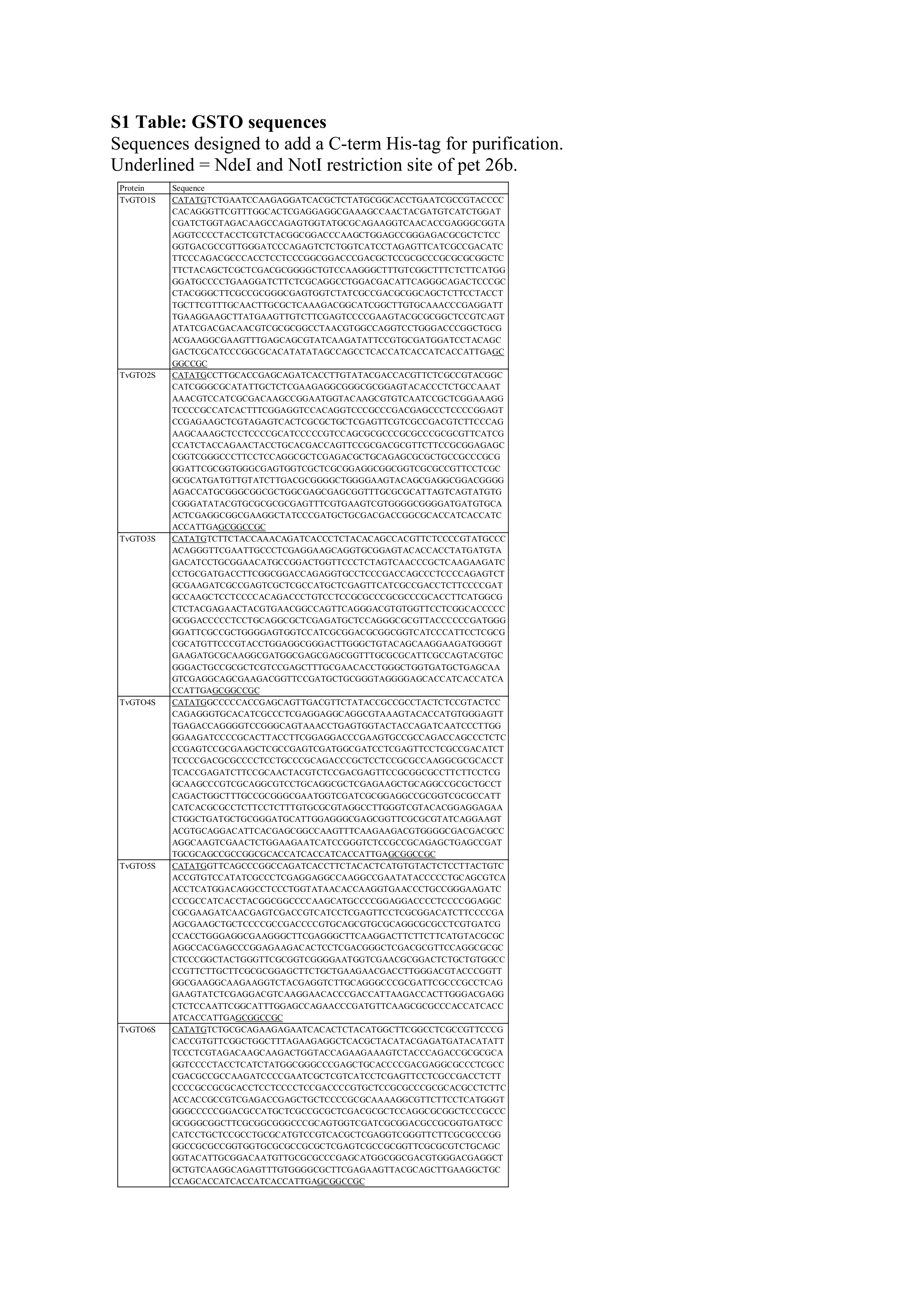

Supplement: S1 Table — Sequences designed to add a C-term His-tag for purification. NdeI and NotI restriction site of pet 26b are underlined. (TIFF) [file pone.0137083.s002.tiff]

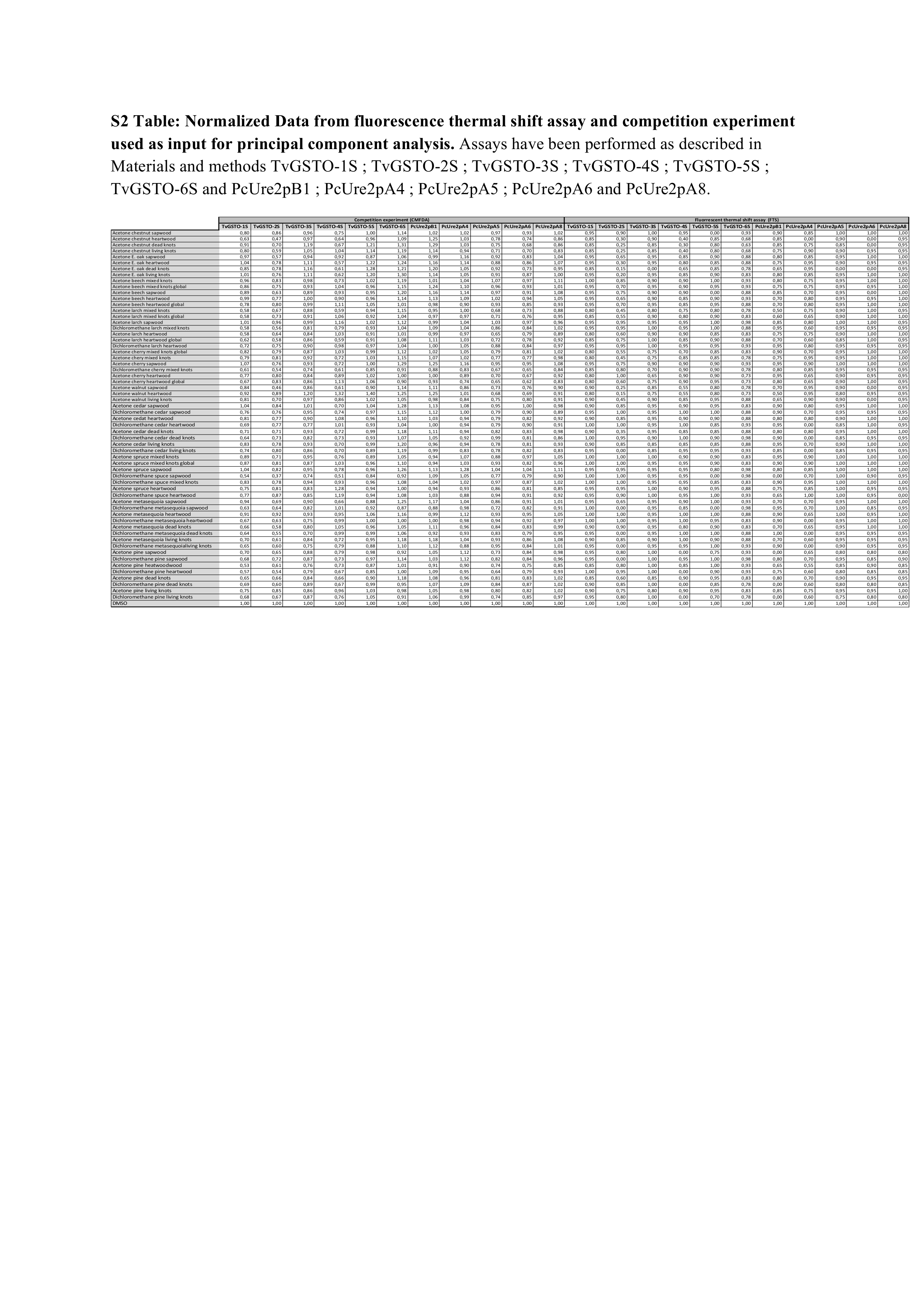

Supplement: S2 Table — Assays have been performed as described in Materials and methods TvGTO-1S; TvGTO-2S; TvGTO-3S; TvGTO-4S; TvGTO-5S; TvGTO-6S and PcUre2pB1; PcUre2pA4; PcUre2pA5; PcUre2pA6 and PcUre2pA8. (TIFF) [file pone.0137083.s003.tiff]

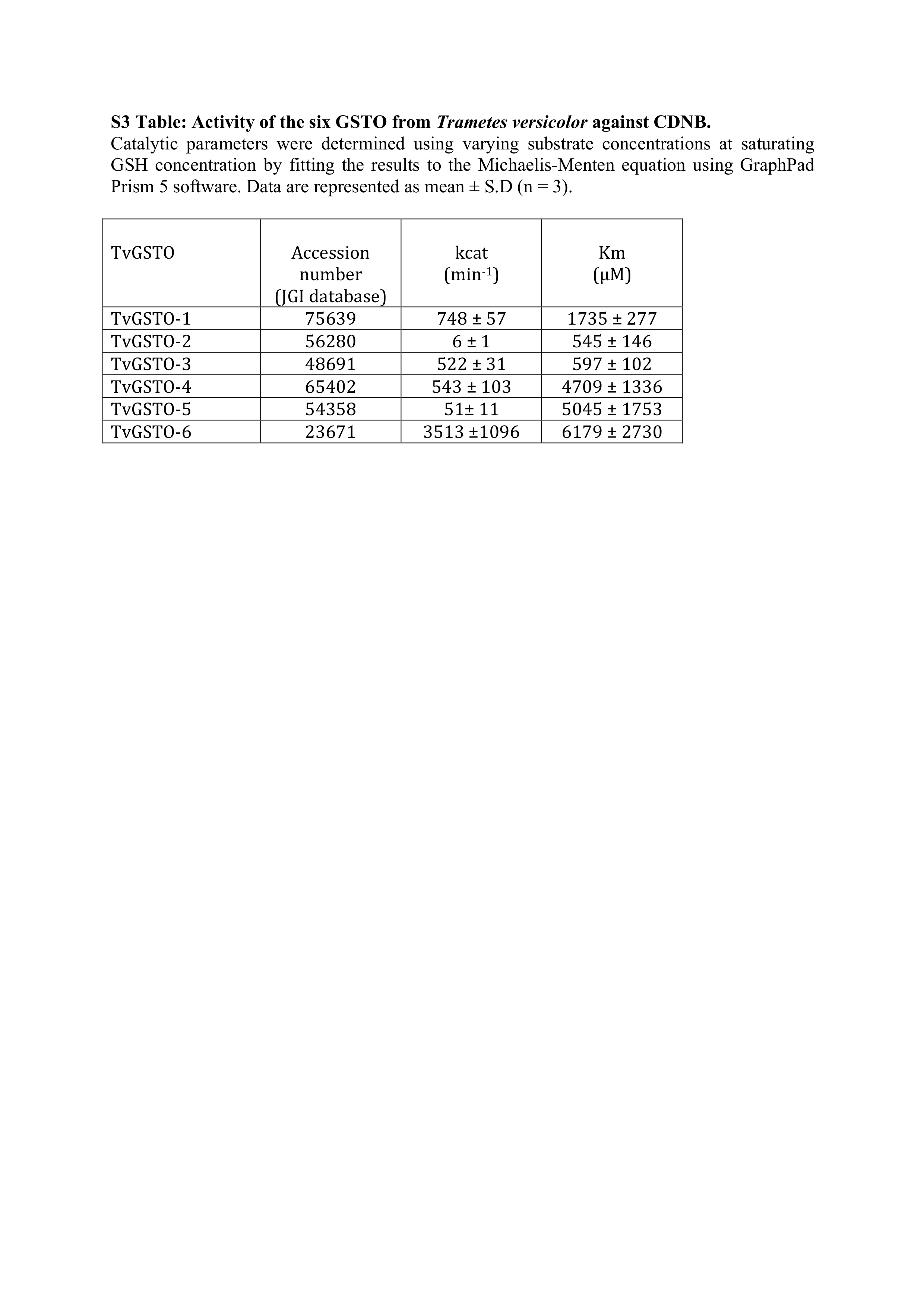

Supplement: S3 Table — Catalytic parameters were determined using varying substrate concentrations at saturating GSH concentration by fitting the results to the Michaelis-Menten equation using GraphPad Prism 5 software. Data are represented as mean ± S.D (n = 3). (TIFF) [file pone.0137083.s004.tiff]

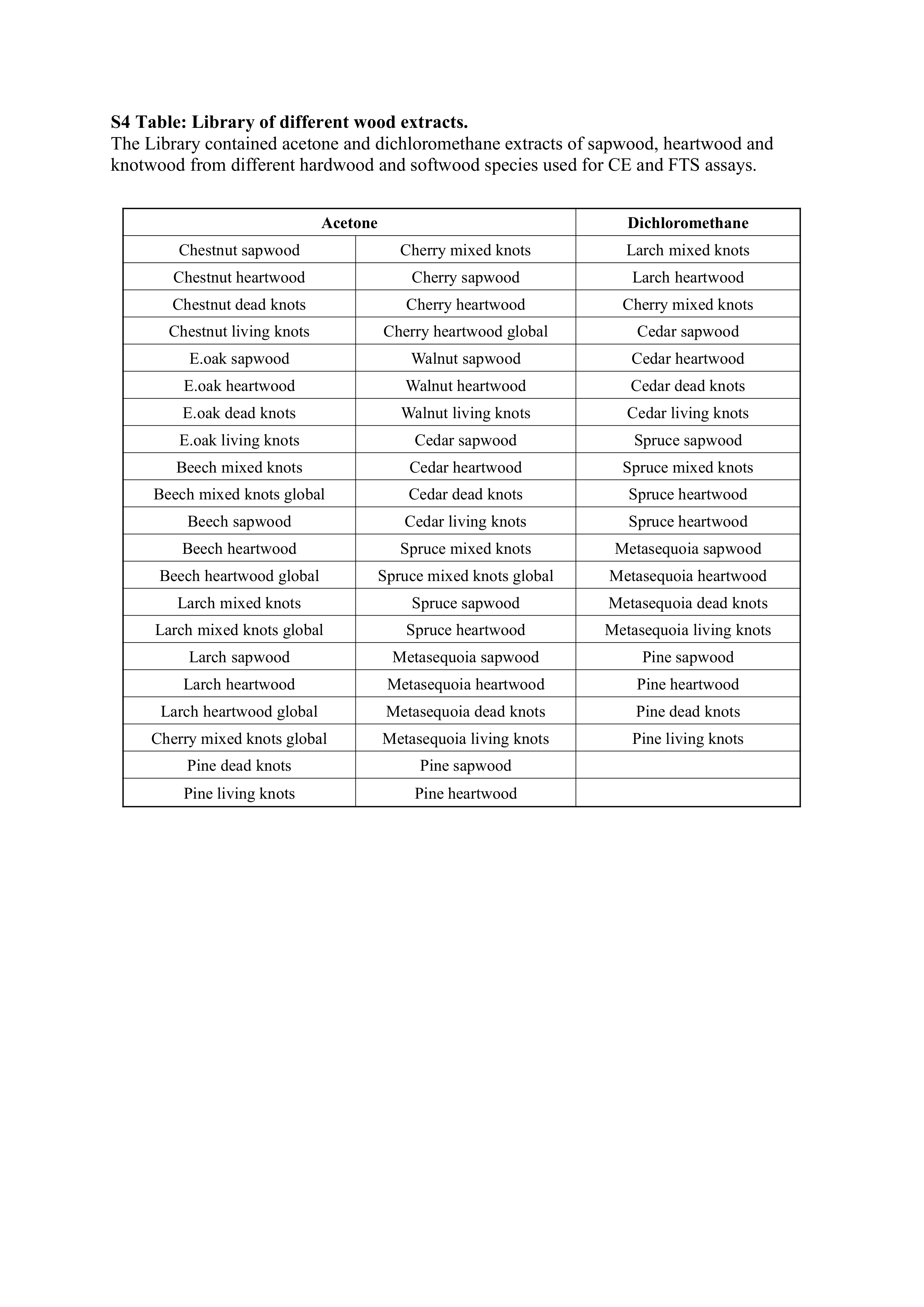

Supplement: S4 Table — The Library contained acetone and dichloromethane extracts of sapwood, heartwood and knotwood from different hardwood and softwood species used for CE and FTS assays. (TIFF) [file pone.0137083.s005.tiff]
